# Early tracheostomy in intensive care trauma patients improves resource utilization: a cohort study and literature review

**DOI:** 10.1186/cc2924

**Published:** 2004-08-23

**Authors:** Yaseen Arabi, Samir Haddad, Nehad Shirawi, Abdullah Al Shimemeri

**Affiliations:** 1 Deputy Chairman, Intensive Care Department (MC 1425), King Abdulaziz Medical City, Riyadh, Kingdom of Saudi Arabia; 2 Associate Consultant, Intensive Care Department (MC 1425), King Abdulaziz Medical City, Riyadh, Kingdom of Saudi Arabia; 3 ICU Pulmonary Fellow, Intensive Care Department (MC 1425), King Abdulaziz Medical City, Riyadh, Kingdom of Saudi Arabia; 4 Chairman, Intensive Care Department (MC 1425), King Abdulaziz Medical City, Riyadh, Kingdom of Saudi Arabia

**Keywords:** intensive care, mechanical ventilation, resource utilization, Saudi Arabia, trauma, tracheostomy, weaning

## Abstract

**Introduction:**

Despite the integral role played by tracheostomy in the management of trauma patients admitted to intensive care units (ICUs), its timing remains subject to considerable practice variation. The purpose of this study is to examine the impact of early tracheostomy on the duration of mechanical ventilation, ICU length of stay, and outcomes in trauma ICU patients.

**Methods:**

The following data were obtained from a prospective ICU database containing information on all trauma patients who received tracheostomy over a 5-year period: demographics, Acute Physiology and Chronic Health Evaluation II score, Simplified Acute Physiology Score II, Glasgow Coma Scale score, Injury Severity Score, type of injuries, ICU and hospital outcomes, ICU and hospital length of stay (LOS), and the type of tracheostomy procedure (percutaneous versus surgical). Tracheostomy was considered early if it was performed by day 7 of mechanical ventilation. We compared the duration of mechanical ventilation, ICU LOS and outcome between early and late tracheostomy patients. Multivariate analysis was performed to assess the impact of tracheostomy timing on ICU stay.

**Results:**

Of 653 trauma ICU patients, 136 (21%) required tracheostomies, 29 of whom were early and 107 were late. Age, sex, Acute Physiology and Chronic Health Evaluation II score, Simplified Acute Physiology Score II and Injury Severity Score were not different between the two groups. Patients with early tracheostomy were more likely to have maxillofacial injuries and to have lower Glasgow Coma Scale score. Duration of mechanical ventilation was significantly shorter with early tracheostomy (mean ± standard error: 9.6 ± 1.2 days versus 18.7 ± 1.3 days; *P *< 0.0001). Similarly, ICU LOS was significantly shorter (10.9 ± 1.2 days versus 21.0 ± 1.3 days; *P *< 0.0001). Following tracheostomy, patients were discharged from the ICU after comparable periods in both groups (4.9 ± 1.2 days versus 4.9 ± 1.1 days; not significant). ICU and hospital mortality rates were similar. Using multivariate analysis, late tracheostomy was an independent predictor of prolonged ICU stay (>14 days).

**Conclusion:**

Early tracheostomy in trauma ICU patients is associated with shorter duration of mechanical ventilation and ICU LOS, without affecting ICU or hospital outcome. Adopting a standardized strategy of early tracheostomy in appropriately selected patients may help in reducing unnecessary resource utilization.

## Introduction

Patients with multiple trauma often require mechanical ventilation for prolonged periods because of their inability to protect their airways, persistence of excessive secretions, and inadequacy of spontaneous ventilation [1]. Tracheostomy plays an integral role in the airway management of such patients, but its timing remains subject to considerable practice variation [2]. The decision to proceed to tracheostomy is often made only if the patient could not be extubated within 10–14 days or more [3]. In 1989, the American College of Chest Physicians Consensus Statement on Artificial Airways in Patients Receiving Mechanical Ventilation considered translaryngeal intubation to be the preferred technique for patients requiring up to 10 days of mechanical ventilation [4]. For those with anticipated need for artificial airway for more than 21 days, tracheostomy was recommended. For all other patients, the decision regarding the timing of tracheostomy was left to daily assessment and physician preference. Such practice was based on earlier reports showing high tracheal stenosis rates with tracheostomy as compared with endotracheal intubation [5,6]. For example, one study reported in 1981 [6] found an incidence of tracheal stenosis after tracheostomy of 65%, as compared with 19% after endotracheal intubation. The authors of that study concluded that tracheostomy for patients requiring an artificial airway for periods as long as 3 weeks could not be recommended. However, the incidence of tracheal stenosis has decreased substantially with recognition of its aetiology and improvements in tracheostomy materials, design and management [7], particularly with the use of high-volume, low-pressure cuffs. Also, the complications associated with prolonged endotracheal intubation are increasingly being recognized, including injury to the larynx and trachea, and patient discomfort. In addition, endotracheal intubation often requires the administration of systemic sedation, with attendant complications. Finally, the incidence of ventilator-associated pneumonia is related directly to the duration of mechanical ventilation [8] – a complication that carries significant morbidity and mortality [9].

One of the under-appreciated consequences of delaying tracheostomy is prolonged mechanical ventilation and intensive care unit (ICU) stay. Notably, the large body of literature addressing local complications of tracheostomy contrasts with the paucity of reports on the advantages of this procedure, especially its impact on resource utilization. This contrast may have encouraged practitioners to consider alternatives to tracheostomy. The aim of the present study is to examine the impact of early tracheostomy on resource utilization in ICU trauma patients. This examination is followed by a review of the existing literature in this area.

## Methods

### Settings

The study was performed at a major tertiary care trauma centre in Riyadh, Saudi Arabia. The 600-bed hospital has a 21-bed medical/surgical ICU staffed by full-time, on-site intensivists 24 hours a day and 7 days a week. Our department has nine consultant intensivists, all of whom are certified in critical care. The hospital has a designated trauma service, including a consultant surgeon, available 24 hours a day. Medical care in the ICU is provided by the ICU team, with the trauma team being responsible for surgical aspects of care. Ventilatory management, and decisions regarding extubation or tracheostomy and discharge from the ICU are made primarily by the ICU team. All percutaneous tracheostomies are performed at the bedside by the ICU team.

### Data collection

We have maintained a prospective database including all consecutive ICU patients admitted since March 1999. For the present study we extracted data on all consecutive patients admitted to the ICU over a 5-year period (March 1999 to February 2004) with new trauma and who underwent tracheostomy during their ICU stay. We excluded patients with history of previous trauma but admitted to the ICU for other reasons, readmissions to the ICU and trauma referrals from other hospitals. Data were collected on demographics and admission severity of illness, estimated using the Acute Physiology and Chronic Health Evaluation (APACHE) II [10], Simplified Acute Physiology Score II [11], postresuscitation Glasgow Coma Score (GCS) and Injury Severity Score (ISS) [12,13]. We documented the presence of injuries to brain, maxillofacial bones, chest, abdominal organs, spinal cord and pelvis/lower extremities. We documented whether an extubation trial was given before tracheostomy. The type of tracheostomy procedure (surgical versus percutaneous) was recorded. The number of days from initiation of ventilation to tracheostomy, from admission to tracheostomy, from tracheostomy to weaning, from tracheostomy to discharge from ICU, the duration of mechanical ventilation, ICU length of stay (LOS) and hospital LOS were all calculated. All these durations were calculated as the number of calendar days, with the day of admission being considered day 0. ICU and hospital mortality rates were documented.

We stratified patients into two groups: the early tracheostomy group, in which tracheostomy was performed within the first 7 days of initiation of mechanical ventilation; and the late tracheostomy group, in which tracheostomy was performed after 7 days. Prolonged ICU stay was defined as ICU stay in excess of 14 days.

### Statistical analysis

Minitab for Windows, release 12.1 (Minitab Inc., State College, PA, USA), was used for statistical analysis. Continuous variables are expressed as means ± standard error of the mean, and were compared using t-tests. Medians and interquartile ranges are also given. Categorical variables are expressed as absolute and relative frequencies, and were compared using χ^2 ^tests. Linear correlation was performed to test for associations between the duration from initiation of mechanical ventilation to tracheostomy and ICU LOS. To assess further the impact of delayed tracheostomy on ICU LOS, univariate and multivariate analyses were performed to examine whether delayed tracheostomy is an independent predictor of prolonged ICU stay. Results of prediction are expressed as odds ratios (ORs) and 95% confidence intervals (CIs). P ≤ 0.05 were considered statistically significant.

## Results

### Baseline patient characteristics

Table 1 summarizes the patients' characteristics at baseline. During the period of study there were 653 trauma admissions to the ICU. The number of patients who required tracheostomy was 136 (21%); 29 patients had tracheostomy within 7 days of mechanical ventilation and the remaining 107 underwent tracheostomy after 7 days. Comparison of demographic data between the two groups revealed no significant differences with regard to age, sex, APACHE II score, Simplified Acute Physiology Score II or ISS. GCS was slightly lower in the early tracheostomy group (5.2 ± 0.5 versus 6.5 ± 0.4; *P *= 0.04). There was no significant difference in the presence of head, chest, abdominal, or pelvic injuries between the groups. Maxillofacial injuries were more common in patients who received early tracheostomy (34% versus 16%; *P *= 0.03) whereas spinal cord injuries were less common (3% versus 16%; *P *= 0.08). The proportions of percutaneous and surgical tracheostomies were not different between the early and late groups.

**Table 1:** Baseline patient characteristics

	Tracheostomy ≤ 7 days	Tracheostomy >7 days	*P*
Number	29	107	
Age (years)	33 ± 3	31 ± 1	0.5
Male sex (%)	26 (90%)	98 (92%)	0.75
APACHE II score	20 ± 1	19 ± 1	0.35
SAPS II score	42 ± 2	39 ± 1	0.36
ISS score	33 ± 2	34 ± 1	0.79
GCS score	5.2 ± 0.5	6.5 ± 0.4	0.04
Type of injury (*n *[%])			
Head	20 (69%)	66 (62%)	0.47
Maxillofacial	10 (34%)	17 (16%)	0.03
Chest	11 (38%)	51 (48%)	0.35
Abdomen	3 (10%)	14 (13%)	0.69
Spinal cord	1 (3%)	17 (16%)	0.08
Pelvic/lower extremities	10 (34%)	40 (37%)	0.77
Percutaneous tracheostomy (*n *[%])	21 (72%)	75 (70%)	0.81

Values are expressed as mean ± standard error of the mean, where appropriate. APACHE, Acute Physiology and Chornic Health Evaluation; GCS, Glasgow Coma Scale; ISS, Injury Severity Score; SAPS, Simplified Acute Physiology Score.

### Tracheostomy timing and main outcomes

Table 2 shows tracheostomy timing data and main outcomes. Extubation trials were performed in 22% of patients with late tracheostomy as compared with 3% of those with early tracheostomy (*P *= 0.019). After placement of the tracheostomy, both groups were weaned off mechanical ventilation and discharged from the ICU after similar periods. Early tracheostomy was associated with a significantly shorter duration of mechanical ventilation (9.6 ± 1.2 days versus 18.7 ± 1.3 days; *P *< 0.0001) and shorter ICU LOS (10.9 ± 1.2 days versus 21.0 ± 1.3 days; *P *< 0.0001). Hospital LOS, ICU mortality and hospital mortality were not different between the two groups.

**Table 2:** Main findings

	Tracheostomy ≤7 days	Tracheostomy >7 days	*P*
Ventilation days before tracheostomy	4.6 ± 0.5 (6, 2.5–7)	13.9 ± 0.5 (13, 10–16)	<0.0001
Days from ICU admission to tracheostomy	4.6 ± 0.5 (6, 2.5–7)	14.1 ± 0.5 (13, 11–17)	<0.0001
Number (%) of patients with extubation trials	1 (3%)	24 (22%)	0.019
Days from tracheostomy to weaning	4.9 ± 1.2 (2, 1–7)	4.9 ± 1.1 (1, 1–4)	1.0
Days from tracheostomy to ICU discharge	6.3 ± 1.3 (4, 2–8.5)	6.9 ± 1.1 (3, 2–7)	0.72
Total duration of mechanical ventilation (days)	9.6 ± 1.2 (8, 6–13)	18.7 ± 1.3 (15, 12–20)	<0.0001
ICU LOS (days)	10.9 ± 1.2 (10, 7–14)	21.0 ± 1.3 (17, 14–23)	<0.0001
Hospital LOS (days)	101 ± 19 (68, 33–139)	105 ± 7 (83, 54–136)	0.84
ICU mortality (*n *[%])	1 (3%)	1 (1%)	NS
Hospital mortality (*n *[%])	5 (17%)	15 (14%)	0.66

Values are expressed as mean ± standard error of the mean (median, interquartile range), where appropriate. ICU, intensive care unit; LOS, length of stay.

**Figure 1:**
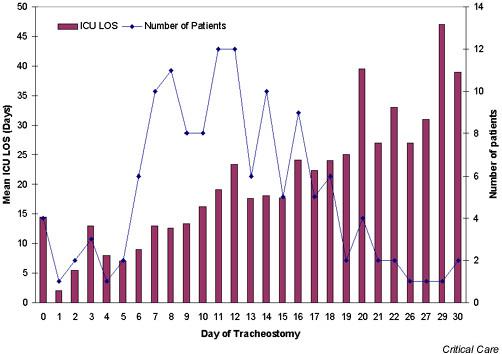
Distribution of patients by timing of tracheostomy and corresponding intensive care unit (ICU) length of stay (LOS). There was a direct correlation between timing of tracheostomy and mean ICU LOS (r = 0.91; *P *< 0.001).

**Figure 2:**
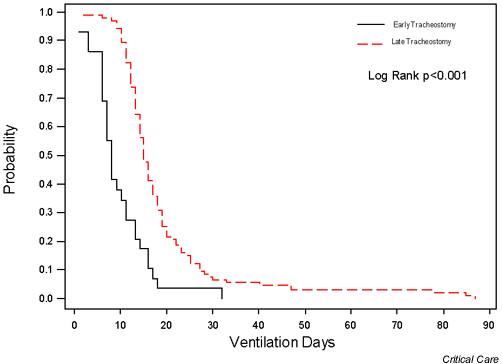
Kaplan–Meier curves of the duration of mechanical ventilation in early and late tracheostomy groups. Early tracheostomy was associated with a significantly shorter duration of mechanical ventilation.

**Figure 3:**
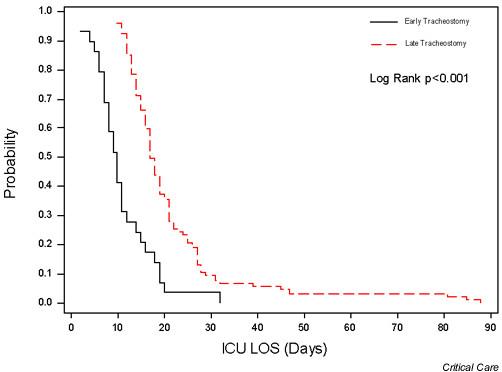
Kaplan–Meier curves of intensive care unit (ICU) length of stay (LOS) in early and late tracheostomy groups. Early tracheostomy was associated with a significantly shorter ICU LOS.

Figure 1 shows the distribution of patients by timing of tracheostomy and the mean ICU LOS for patients, stratified by timing of tracheostomy. There was a direct correlation between the timing of tracheostomy and mean ICU LOS (r = 0.91; *P *< 0.001). Figures 2 and 3 show Kaplan–Meier curves of the duration of mechanical ventilation and ICU LOS in the two groups. Similarly, both the duration of mechanical ventilation and ICU LOS were significantly shorter in the early tracheostomy group (log rank *P *value < 0.001 for both).

Using univariate analysis the following factors were found to be associated with prolonged ICU stay (>14 days): late tracheostomy (OR 7.7, 95% CI 3.0–19.9; *P *< 0.001), spinal cord injury (OR 6.1, 95% CI 1.3–27.7; *P *= 0.019) and extubation trials (OR 3.1, 95% CI 1.1–8.7; *P *= 0.037). The presence of head injury was a significant negative predictor of prolonged ICU stay (OR 0.5, 95% CI 0.2–1; *P *= 0.047), as was the presence of maxillofacial bone injuries (OR 0.4, 95% CI 0.2–1.01; *P *= 0.052). APACHE II score, ISS and GCS score exhibited no significant association with prolonged ICU stay. Using multivariate analysis, late tracheostomy (OR 6.9, 95% CI 2.6–18.1; *P *< 0.001) and, to a much lesser extent, spinal cord injury (OR 4.7, 95% CI 0.99–22.6; *P *= 0.052) emerged as independent predictors of prolonged ICU stay.

## Discussion

In our study we found that early tracheostomy in trauma ICU patients was associated with a significant reduction in the duration of mechanical ventilation and ICU LOS without affecting patient outcome. Weaning patients from mechanical ventilation and discharge occurred shortly and in similar periods after tracheostomy in both groups, suggesting that tracheostomy was a critical factor in weaning and discharge. We also found that late tracheostomy was an independent predictor of prolonged ICU stay.

The study also showed that tracheostomy was more likely to be performed early in patients with maxillofacial fractures, reflecting the need for this procedure for airway management. In patients with spinal cord injury tracheostomy was more likely to be performed late because many of these patients had to undergo surgical spinal fixation before tracheostomy. In such cases, the surgeons preferred to wait until the surgical wound in anterior spinal fusion was healed before performing the tracheostomy. Patients with early tracheostomy had lower GCS, reflecting the common practice of performing tracheostomies earlier in patients with low GCS while delaying tracheostomy in patients with higher GCS in case extubation becomes possible.

The very low mortality seen in the patients we studied may be explained by selection of proper candidates for tracheostomy, excluding those patients who were unlikely to survive. Hospital LOS in these patients was prolonged, reflecting their severe injuries that required lengthy rehabilitation periods. The very limited rehabilitation facilities meant that the patients had to undergo rehabilitation while they were hospitalized, prolonging further the hospital LOS.

Table 3 summarizes studies that examined the impact of early tracheostomy on resource utilization [2,3,14-18]. All of these studies, except one [2], found reduction in the duration of mechanical ventilation, ICU LOS and/or hospital LOS. Some of these studies found reduction in ventilator-associated pneumonia or colonization incidence. Some of the studies [3,14-16,18] were retrospective, and all found a positive impact of early tracheostomy on duration of mechanical ventilation, ICU LOS, hospital LOS, or pneumonia rates. The study by Rodriguez and coworkers [17] was a prospective randomized trial in which patients were assigned to early tracheostomy (≤7 days) if they were admitted on an odd day and to late tracheostomy if admitted on an even day. That study found a reduction in duration of mechanical ventilation, ICU LOS and hospital LOS. Sugerman and coworkers [2] conducted a 'multicenter' randomized trial in five centres involving patients with head trauma, nonhead trauma and no trauma. Those investigators randomized patients on days 3–5 to receive tracheostomy or to continue with translaryngeal intubation. A second randomization for patients who remained intubated was performed on days 10–14. Those authors found no differences in ICU LOS or frequency of pneumonia between early and late tracheostomy. However, the study had several limitations. Out of the five participating centres, only one completed the study. Out of 157 eligible patients, only 112 completed the study because of physician bias and incomplete information. Only 14 patients entered the second randomization. That report illustrates the difficulty in performing studies that challenge widely accepted beliefs. Reviewing these studies also illustrates the lack of consensus regarding the definition of early tracheostomy, with different cutoff points used ranging between 3 and 14 days.

**Table 3:** Literature review

Ref.	Type of study	Number of patients	Reason for admission	Timing of tracheostomy	Main outcomes
[3]	Retrospective	101	Blunt multiple trauma	Early tracheostomy ≤4 days Late Tracheostomy >4 days	↓Duration of MV, ↓incidence of nosocomial pneumonia
[14]	Retrospective	31	Head trauma	Early tracheostomy ≥7 days Late tracheostomy >7 days	↓Duration of MV, ↓hospital LOS, ↓ICU LOS
[15]	Retrospective	118	Multiple trauma	Early tracheostomy ≤3 days Intermediate tracheostomy 4–7 days Late tracheostomy >7 days	↓Incidence of pneumonia
[18]	Retrospective	157	Blunt trauma	Early tracheostomy ≤6 days Late tracheostomy >6 days	↓Duration of MV, ↓ICU LOS, ↓hospital LOS, ↓hospital charges
[16]	Retrospective	30	Neurosurgical (CVA, head injury, trauma, infection)	Early tracheostomy ≤7 days Late tracheostomy >7 days	↓Duration of MV, ↓incidence of colonization, ↓faster recovery from pneumonia
[17]	Prospective randomized	106	Multiple trauma	Early tracheostomy ≤7 days Late tracheostomy >7 days	↓Duration of MV, ↓ICU LOS, ↓hospital LOS, ↓pneumonia if tracheostomy was performed earlier than 3 days
[2]^a^	Prospective randomized multicentre	157 eligible patients	Head-trauma, Nonhead trauma, no trauma	First randomization: 3–5 days Second randomization: 10–14	No difference in ICU LOS, frequency of pneumonia, or death

^a^Of five participating centres, only one completed the study; of 157 eligible patients, only 112 completed the study because of physician bias and incomplete information; and only 14 patients entered the second randomization. ICU, intensive care unit; LOS, length of stay; MV, mechanical ventilation.

Strengths of our study include prospective data collection ensuring complete data and the relatively large number of patients. However, data extraction and analysis was retrospective. Because the database was not designed specifically to examine tracheostomy practices, certain issues were not documented, such as when the decision for tracheostomy was made and how different intensivists and surgeons varied in their timing of tracheostomy. In addition, the study was observational and was conducted from one centre. A large multicentre randomized controlled trial in which patients are randomized to early versus late tracheostomy would be the ideal way to test the impact of procedure timing on resource utilization.

In summary, the present study, in addition to the existing literature, indicates that early tracheostomy is associated with reduced ICU LOS. Adopting a standardized strategy may help in improving resource utilization. In addition, there is an urgent need for a multicentre randomized controlled trial to assess the most appropriate timing for tracheostomy.

## Key messages

• Early tracheostomy in trauma ICU patients was associated with shorter duration of mechanical ventilation and ICU LOS without affecting ICU or hospital outcomes.

• There was a direct correlation between timing of tracheostomy and ICU LOS.

• Using multivariate analysis, late tracheostomy emerged as an independent predictor of prolonged ICU LOS.

## Competing interests

None declared.

## Abbreviations

APACHE = Acute Physiology and Chronic Health Evaluation; CI = confidence interval; ICU = intensive care unit; ISS = Injury Severity Score; GCS = Glasgow Coma Score; LOS = length of stay; OR = odds ratio.
